# Methyl-Lysine-Dependent Control of Protein Lifetime Through Lysine-Node Crosstalk and Reader-Coupled Degradation

**DOI:** 10.3390/biology15040330

**Published:** 2026-02-14

**Authors:** Brad E. Morrison

**Affiliations:** 1 Department of Biological Sciences, Boise State University, Boise, ID 83725, USA; bradmorrison@boisestate.edu; 2 Biomolecular Sciences Graduate Program, Boise State University, Boise, ID 83725, USA

**Keywords:** protein methylation, lysine methylation, proteostasis, ubiquitin, degrons, autophagy, chaperones, epigenetics

## Abstract

The half-life of proteins is determined by the “information” carried within specific regions of the protein. Lysine methylation is an emerging post-translational modification that can act at the decision node. In contrast to the charge-neutralizing effects of lysine acylation, methylation maintains the positive charge of the lysine residue, allowing for the presence of multiple methylation states (Kme1/2/3). This property enables the “reading” of the methylation state by “methyl lysine readers.” Although the methyl group is chemically stable, the methylation is enzymatically reversible by lysine demethylases. In this review, we conceptualize the “methyl lysine” as an “information”-containing “signal” at the decision node. The “information” is composed of the interplay between the “competition” among the same site modifications, the “methylation-dependent degron” or “methyl degrons,” the “steric blockade” or “methyl shields,” and the “changes in spatial routing” or “methyl routing cues.” In this review, we discuss the emerging evidence within these three types of functional methyl lysine.

## Simple Summary

Cellular proteins are constantly being made and broken down. When the rate of breaking down proteins is too high, cells may lose important functions. On the other hand, if the process of breaking down proteins is impaired, proteins may accumulate or be mislocalized. Proteins are composed of various regions that determine the destiny or the “decision node” of the protein. In this review, we discuss the evidence for lysine methylation as an important post-translational modification at the decision node, which is long-lived, enzymatically reversible, and changes the “reading” of the protein by the cell. The three types of functional methyl lysine are “methyl degrons,” “methyl shields,” and “methyl routing cues,” and we outline the experiments needed to determine which type is involved with each protein.

## 1. Introduction

The half-lives of most proteins are precisely regulated in relation to their cellular functions. Proteins may have short half-lives, which are essential for efficient signal transduction, while others may have longer half-lives, which are important for structural integrity. This necessitates a hierarchical structure of degradation and decision-making systems, which may include the ubiquitin–proteasome pathway (UPS) [1], lysosomal and autophagic systems, and molecular chaperones. One common feature of these systems is that degradation decisions are often not based solely on the protein structure but, in many cases, are dictated by small regions that may function as “decision nodes” where post-translational modifications, protein–protein interactions, and structural changes may determine the fate of a protein, either in terms of degradation, recycling, or stabilization. Among post-translational modifications, lysine methylation has traditionally been implicated in chromatin regulation, although large-scale proteomics and functional analyses have revealed that many non-histone proteins are also targets of methylation, and these modifications may regulate various cellular processes, including protein stability, localization, and interaction profiles [2,3,4,5,6]. Lysine methylation, being a relatively stable post-translational modification (PTM), may function as a “memory” mark in cellular regulation. Nevertheless, lysine methylation is not an irreversible PTM event, as lysine methyltransferases (KMTs) are responsible for lysine methylation, while lysine demethylases (KDMs), which may include flavin-dependent KDMs like KDM1/LSD1 and JmjC domain-containing KDMs, are involved in the removal of these modifications, thereby enabling dynamic regulation of methylation status and occupancy. Importantly, methylation of lysine residues in a protein does not confer any intrinsic resistance against proteolysis; rather, these modifications may modulate proteolytic handling by cellular systems.

## 2. Lysine Decision Nodes as Protein-Lifetime Control Points

### 2.1. Decision Nodes: Single-Residue “Switches” Versus Clustered “Cassettes”

Decision nodes can be as simple as a single critical lysine residue, which is repeatedly targeted for modification, or as complex as a localized “cluster” of lysine residues that collectively contribute to protein recognition. In the single-residue model, a single critical lysine residue can act as a “switch” with two or more states. The modification of this “residue can create or eliminate a protein–protein interface, such as between a reader and a ligase, or regulate access to a “degron” or modulate protein–protein interactions in an abrupt fashion. In the cluster or cassette model, a localized cluster of lysine residues acts as a cassette of acceptor sites for protein modifications. Individual sites within this cassette can be partially redundant, such that PTM patterning or graded changes in charge or hydrophobicity can be used to integrate information at this node. The single-residue model is most appropriate when a binding interface or catalytic requirement selects for modification of a specific lysine residue. The cluster model is more appropriate when protein regulation depends on the overall density of protein modifications within this cluster or cassette of sites.

### 2.2. Routing Labels Versus Routing Consequences

Two terms are often used synonymously, but are distinguished in the following. A routing label refers to the cue provided by the PTM, as well as the interactions enabled by the PTM with the reader/effector. A routing consequence refers to the outcome that follows as a result such as proteasomal degradation, sequestration by autophagosomes, retention in the nucleus, or partitioning into membraneless compartments [7]. This review focuses on the role of methyl-lysine as a routing label at decision points, as well as how the outcome of the routing consequence might be experimentally ascertained.

### 2.3. Overview of Methyl-Lysine Decision-Node Mechanisms

Methyl-lysine decision nodes can be classified into three functional groups: (i) methyl degrons, in which methylation is required for recognition by a reader-E3 complex to facilitate ubiquitination-mediated degradation; (ii) methyl shields, in which methylation inhibits degradation, either by steric hindrance or by attracting inhibitory proteins; and (iii) methyl routing cues, in which methylation influences subcellular location or complex membership, thereby indirectly affecting degradation rates. While these groups are distinct, they can be dynamic, with each node potentially shifting among groups as additional PTMs are added.

## 3. Methylation as an Information-Rich Modification

### 3.1. Chemical and Enzymatic Features of Lysine Methylation

Unlike acetylation or other lysine acylations, methylation of lysine preserves the residue’s original charge while modulating sterics and hydrophobicity. Lysines can be mono-, di-, or trimethylated, and many methyl-lysine “reader” domains, including Tudor, MBT, chromo, and PHD domains, can recognize each of these states, at least in part, due to the geometry of aromatic “cages” that recognize these marks [8,9]. Methylation is stable under physiological conditions, but its presence is enzymatically labile, as flavin-containing and JmjC-containing lysine demethylases (KDMs) can remove methyl groups from lysines, often showing specificity for certain substrates or for the presence of specific numbers of methyl groups [10]. This reversibility is critical for the logic of decision nodes, in that the presence of methylation can be dynamic, responding to cellular needs rather than being an unchangeable “modification”.

### 3.2. Measuring Methylation Occupancy at Decision Nodes

However, a limitation in interpreting the functional role of methyl lysine is that the majority of the data provide information on the presence of methylation sites without quantitation. A lysine that is only methylated in 1–5% of the population can still be functional if it has high-affinity readers or if it nucleates multivalent interactions. However, the understanding of the mechanism can be refined if the occupancy is quantitatively measured. Quantitative proteomics can be employed to estimate the occupancy, while the ubiquitin Kε-GG can be coupled to determine if methylation is associated with local ubiquitination at the same lysine [11,12].

## 4. Same-Site Competition Among Lysine Modifications

Lysine is a hub for different PTMs, including ubiquitination, acetylation, SUMOylation, and different acylations. When two PTMs compete for the lysine, the decision node can be a competitive switch, where the binding of one PTM inhibits the binding of the other PTM. Acetylation can inhibit ubiquitination, thereby protecting the proteins from degradation. On the other hand, ubiquitination can compete with acetylation for the lysine, thereby promoting degradation [13]. The same logic can be applied to methylation and ubiquitination, where methylation inhibits the binding of ubiquitin. This can be exemplified in the case of acetylation and ubiquitination competing in the regulation of the stability of MCL1 and other proteins. Therefore, the same-site competition for lysine should be tested when methylation is implicated in the regulation of degradation [14,15].

## 5. Methyl-Degrons: Methylation-Enabled Recruitment of Ubiquitin Ligases

In the case of methyl-degrons, the decision node is a PTM that recruits an E3 ligase via a methyl lysine reader. The methylation-dependent PTM is the only PTM that recruits the E3 ligase. Therefore, in the case of methyl-degrons, methylation is not only permissive; it is the only PTM that enables the binding of the E3 ligase.

### 5.1. Reader-E3 Coupling and Substrate Selection

There are a number of methyl-lysine reader families that have been shown to link methylated substrates with ubiquitination machinery. One well-characterized example is that of the methyl-lysine reader protein L3MBTL3, which has been shown to link with a CRL4^DCAF5^ E3 ligase complex to target methylated substrates for degradation. For example, methylation events mediated by SETD7 have been shown to create a link that facilitates ubiquitination of certain target proteins [16,17].

### 5.2. A Methyl-Degron Framework for Discovery

A useful criterion for the identification of methyl-degrons involves testing whether (i) methylation increases ubiquitination on or near the node, (ii) inhibition of the proteasome stabilizes the protein, and (iii) elimination of the KMT or the reader abolishes ubiquitination and stabilizes the protein. In contrast, forced methylation should destabilize the protein. Recent reviews provide discovery frameworks for additional methyl-degrons on non-histone proteins based on the emerging mechanisms of methylation-dependent proteolysis [18,19].

## 6. Methyl-Shields: Methylation-Mediated Protection from Degradation

Decision nodes classified as methyl-shields contain methylation events that protect from degradation. These may be mediated through steric blockage of ubiquitin attachment on the protein, exclusion of degron recognition by other degrons, and/or protection through the action of deubiquitinating enzymes (DUBs), chaperones, and/or scaffold proteins. In this case, methylation serves as a stabilizer.

### 6.1. Steric Blockade and Same-Site Exclusion of Ubiquitination

If methylation occurs on the same residue as the target for ubiquitin attachment, it may serve as a cap that excludes ubiquitin attachment and favors protein stability. Although this mechanism has intuitive appeal, it remains important to demonstrate that the same residue is used for ubiquitin attachment under physiological conditions.

### 6.2. Reader-Mediated Shielding and Recruitment of Protective Factors

Readers may protect from degradation through the recruitment of protective factors that counteract ubiquitin attachment and/or unfoldase activity. These protective factors may be involved in the shielding action through the occlusion of degrons and/or the action of DUBs.

### 6.3. Methyl-Shield Diagnostics

Key experiments include identifying lysine(s) that become methylated, assessing K→R or K→A mutations, measuring changes in ubiquitination when methylation is inhibited, assessing if proteasome and lysosome/autophagy inhibitors differentially affect protein stability, and identifying reader or partner proteins that depend on methylation. In addition, measuring changes in substrate solubility, aggregation propensity, and association with molecular chaperones can help differentiate between shielding and other effects on protein folding or targeting.

### 6.4. Limitations and Gaps for Methyl-Shields

The methyl-shield concept is compelling, although it remains to be fully understood. Key gaps include: (i) a lack of quantitative methylation occupancy at sites where shields might act, (ii) a lack of knowledge regarding reader domains that function as stabilizers rather than as part of a degradative complex in non-histone contexts, (iii) a lack of knowledge regarding how methylation might compete with multiple lysine modifications and ubiquitin-like protein additions at a site, and (iv) a lack of knowledge regarding how methylation might affect changes in local ubiquitin chain types, DUB engagement, or unfoldase engagement. These gaps can be filled by integrating occupancy-aware methyl-proteomics, ubiquitin chain analysis, and reader-focused interactomics under defined cellular states.

## 7. Methyl-Routing Cues: Methylation-Driven Changes in Spatial Fate and Complex Membership

Routing cues define decision points where methylation controls changes in protein subcellular location or complex membership, thus affecting protein half-life. Routing can affect access to proteolytic systems, induce condensate formation, or affect engagement with autophagy receptors. Autophagy receptors, such as p62, integrate ubiquitin-dependent cues to route substrates into lysosomal degradation pathways [20].

### 7.1. Localization Shifts and Access to Degradation Systems

Localization is an important regulator of lifetime because the degradation machinery is compartmentalized. Methylation may create or destroy binding sites for transport proteins or scaffolds, thereby modulating the proportion of the substrate accessible to the UPS or lysosomal pathways. An example includes methylation events that modulate nuclear/cytoplasmic localization and chromatin binding activities in non-histone proteins [5,21]. Compartmentalized methylation events are also known to occur in the mitochondria, which may affect the function and regulation of mitochondrial proteins [22].

### 7.2. Complex Membership and Phase-Separated Environments

In other instances, methylation may act as a routing label by modulating complex membership, including the presence or absence of RNA-binding proteins. The partitioning of proteins into condensates [23] may either protect the protein from degradation or concentrate it with the degradative machinery, depending on the composition of the organelles or the presence or absence of cellular stress. The reader domain specificity and multivalency may be important regulators in such processes [21,24,25,26].

### 7.3. Routing Diagnostics: Separating Label from Consequence

In assessing the role of methylation in the regulation of protein degradation, it is important to evaluate the localization, complex membership, and the lifetime within each compartment. It is important to note that the methylation may affect the localization or complex membership (label) or the subsequent degradation or stability (consequence) of the target protein. The distinction between the label and the consequence is important to avoid the common mistake of observing methylation with changes in protein stability without the methylation directly engaging the degradative machinery.

## 8. Pathological Dysregulation of Methyl-Lysine Decision Nodes

The decision node logic makes the regulation of the methylation pathways highly susceptible to changes in the levels or activities of the writers, erasers, or the levels or activities of the reader proteins. The changes may lead to the aberrant regulation of the target proteins, resulting in the misrouting of the target proteins. In cancer or neurodegeneration, the decision node logic may be important in the regulation of the proteostasis pathways.

### 8.1. Cancer

Cancer often manifests through the dysregulation of methylation enzymes and reader networks. Non-histone lysine methylation has the potential to modulate the stability and activity of tumor suppressors and oncogenic regulators, which might be mediated through methyl-degrons or methyl-shields. Recent reviews emphasize the importance of the KMT/KDM system in growth factor signaling, DNA damage response, and the regulation of transcription factors, thus confirming the potential of the system to act as a biomarker and therapeutic target, particularly through the modulation of the stability of various proteins, such as tumor suppressors and oncogenic regulators [27,28]. The regulation of the stability of two important proteins, Mdm2 and MdmX, is a good example of the tremendous potential of the system to modulate the stability of proteins very quickly, particularly in the context of oncogenesis [29].

### 8.2. Neurodegeneration

Neurodegenerative diseases are associated with the dysregulation of proteins and the cellular stress response. Lysine methylation is implicated in the regulation of gene expression in neurons and might also modulate the methylation of non-histone proteins, which might be associated with proteostasis. Recent syntheses suggest that the dysregulation of methylation networks might modulate the stability of proteins associated with the survival and aggregation of neurons, although the decision nodes of the system are not well elucidated and represent a significant opportunity for future research [30].

### 8.3. Translational Opportunity

From a translational point of view, the decision nodes of the methyl-lysine system represent a significant opportunity, particularly because of the potential to target the system through various approaches, from the inhibition or activation of the KMT/KDM system to the inhibition of the reader domains and the modulation of the competing PTMs. The determination of the causal relationships between methylation, the reader domains, the routing label, and the consequence is a very significant challenge.

## 9. Methods for Detecting Methyl-Lysine Decision-Node Behavior

### 9.1. Summary of Experimental Approaches

The mechanistic assignment can be improved with site-mapping, quantification of methylation states, ubiquitin profiling, and interaction analyses. Table 1 lists some methodologies that can be used for discriminating methyl-degrons, methyl-shields, and methyl-routing cues.

### 9.2. Diagnostic Panel and Interpretability

**Figure 1:**
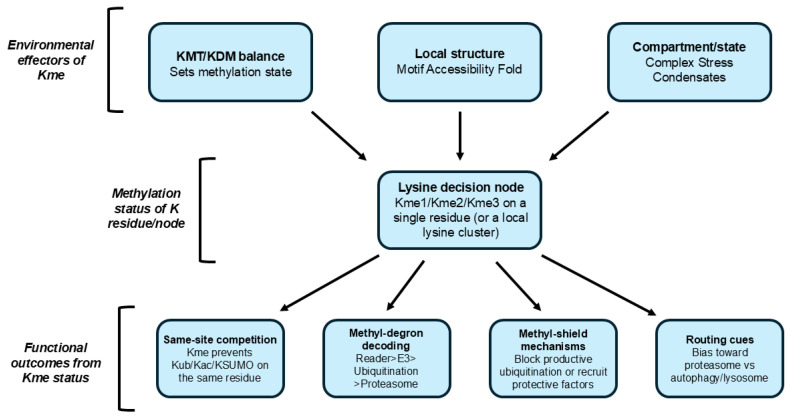
A “lysine decision node” model linking lysine methylation to protein fate.

Figure 1 represents a model for cellular environment, Kme status and functional outcomes. The methylation status of the lysine residue is influenced by three main factors: (i) the balance of lysine methyltransferases and demethylases, (ii) the intrinsic accessibility of the lysine, and (iii) the cellular environment of the protein, including whether it is part of a complex, under stress, or within a condensate. These factors combine to result in an unmethylated, Kme1, Kme2, or Kme3 residue status, respectively. Significantly, this status is dynamic, meaning that changes in the levels of enzymes, their subcellular location, as well as small changes in the structure of the proteins, can result in the exposure or sequestering of the lysine residue. The subcellular environment is important because certain environments can be rich in enzymes and binding partners that facilitate methylation, while others can be less favorable for methylation of the residue.

Once methylation is added, it can affect protein fate in a variety of ways. First, methylation can compete with other modifications, such as ubiquitination, and, in some cases, SUMOylation, based on site occupancy. Second, methylated lysines can act as a signal through reader protein binding, potentially acting as a degron to recruit the ubiquitin–proteasome pathway and induce rapid protein degradation. Third, methylation can stabilize a protein by acting as a methyl shield, inhibiting ubiquitination or recruiting a stabilizing protein that enhances solubility. Finally, methylation can affect protein fate by affecting routes of degradation, targeting a protein to the proteasome or autophagy/lysosome pathways, a distinction that is important when a protein is part of a complex, condensate, or aggregated state.

The methylation state of a specific lysine residue (Kme0/1/2/3) is regulated by the cellular/environmental context, including the levels of methylating and demethylating enzymes (KMT/KDM), structural factors, and compartmental/state effects. The methylation state of a specific lysine residue (Kme0/1/2/3) may facilitate functional outcomes that: (i) compete with other lysine modifications such as ubiquitination or SUMOylation; (ii) be recognized as a methylated degron by reader proteins; (iii) stabilize the protein through methyl shield effects inhibiting ubiquitination or activating protective factors; and (iv) direct the clearance pathway to proteasomal degradation or the autophagy–lysosome pathway.

## 10. Conclusions

The literature supports a unifying model that methyl-lysine conveys decision-node information read by reader domains that interact with proteostasis pathways. The decision-node model focuses attention on mechanistic diagnostics, occupancy, reader coupling, competition between modifications at the same site, and compartment/state turnover rather than relying on a relationship between methylation levels and protein abundance.

Lysine residues have several acyl modifications other than acetylation, including lactylation, propionylation, and butyrylation. These modifications often involve changes in charge or large chemical groups that are related to cellular metabolism. While some decision-node mechanisms may apply across various lysine PTMs, methylation has a unique ability to impart a positive charge that also enables a multi-state readout through Kme1/2/3 and reader proteins [8,9,31]. The integration of methylation with other crosstalk between lysine PTMs, particularly other decision nodes outside histones, will be an interesting area of research [32,33].

The next steps will involve: (i) occupancy-based methyl proteomics under relevant physiological conditions; (ii) reader-E3 ligase complexes vs. reader-protective complexes in non-histone decision nodes; (iii) clear distinction between routing labels and outcomes; and (iv) disease-based mechanistic validation of decision nodes. Future research will highlight whether methyl-lysine functions as a biomarker or a target for intervention.

## Figures and Tables

**Table 1:** Representative experimental approaches for diagnosing methyl-lysine decision-node mechanisms.

Mechanism Class	Molecular Mechanism	Deterministic Approaches	References
Same-site competition	Competition among mutually exclusive lysine PTMs at a decision node alters lifetime.	Site-directed mutagenesis; PTM-specific enrichment; ubiquitin vs methyl occupancy measurements.	[11,13,15,31]
Methyl-degron	Methylation creates a reader-E3 ligase interface, increasing ubiquitination and degradation.	KMT/KDM perturbation; reader/E3 knockdown; ubiquitin chain profiling; proteasome inhibition; half-life assays.	[14,17,18]
Methyl-shield	Methylation blocks ubiquitination or recruits protective partners, stabilizing the protein.	Same-site ubiquitin competition tests; reader mapping; DUB/chaperone association; compartment-specific turnover assays.	[15,21,31]
Methyl-routing cue	Methylation changes localization or complex membership, indirectly changing access to degradation.	Imaging/fractionation; interactomics; compartment-specific turnover; autophagy/lysosome vs proteasome inhibitors.	[20,22,23,24]

## Data Availability

No new data were created or analyzed in this study. Data sharing is not applicable to this article.

## Abbreviations

AAA+	ATPases associated with diverse cellular activities
CRL	Cullin-RING ligase
DCAF	DDB1-CUL4 associated factor
DUB	deubiquitinating enzyme
JmjC	Jumonji C (domain family)
KDM	lysine demethylase
KMT	lysine methyltransferase
Kme1/2/3	mono-/di-/tri-methyl lysine
PTM	post-translational modification
UPS	ubiquitin–proteasome system
